# Supplementary figures and images for: Artificial Intelligence-Enhanced Quantitative Ultrasound for Breast Cancer: Pilot Study on Quantitative Parameters and Biopsy Outcomes
Source: Diagnostics (Basel). 2024 Feb 14;14(4):419. doi: 10.3390/diagnostics14040419 (PMC10888332; doi:10.3390/diagnostics14040419)

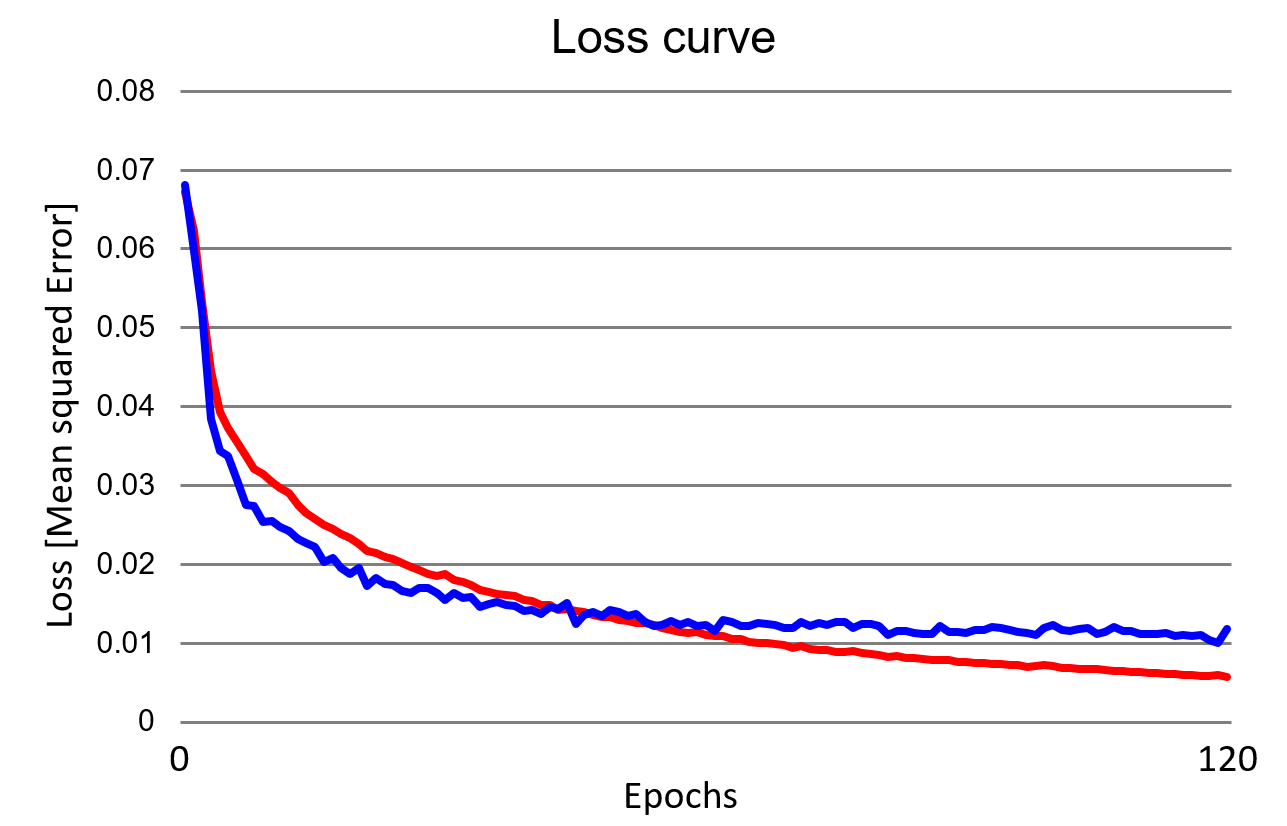

Supplement: Supplementary file 1 [file diagnostics-14-00419-s001.zip › diagnostics-2865301-supplementary.png]
